# Micro/Nanoplastic Exposure on Placental Health and Adverse Pregnancy Risks: Novel Assessment System Based upon Targeted Risk Assessment Environmental Chemicals Strategy

**DOI:** 10.3390/toxics12080553

**Published:** 2024-07-30

**Authors:** Danyang Wan, Yujie Liu, Qianjing Chang, Zhaofeng Liu, Qing Wang, Rui Niu, Beibei Gao, Quanquan Guan, Yankai Xia

**Affiliations:** 1 Department of Pathology and Pathophysiology, School of Medicine, Southeast University, Nanjing 210009, China; wandy220284@163.com; 2 State Key Laboratory of Reproductive Medicine, School of Public Health, Nanjing Medical University, Nanjing 211166, China; yujieliu@stu.njmu.edu.cn (Y.L.); nj20210129@163.com (Q.C.); liuzhaofeng@stu.njmu.edu.cn (Z.L.); wangqing@stu.njmu.edu.cn (Q.W.); nrnr7516@sina.com (R.N.); gaob@njmu.edu.cn (B.G.); 3 Key Laboratory of Modern Toxicology of Ministry of Education, School of Public Health, Nanjing Medical University, Nanjing 211166, China

**Keywords:** risk assessment, TRAEC strategy, micro/nanoplastic, reproductive toxicity, placenta

## Abstract

Micro/nanoplastics (MNPs), as emerging pollutants, have been detected in both the maternal and fetal sides of the placenta in pregnant women, and their reproductive toxicity has been demonstrated in in vivo and in vitro experimental models. The Targeted Risk Assessment of Environmental Chemicals (TRAEC) strategy has been innovatively devised to facilitate valid risk assessment, encompassing a comprehensive evaluation of reliability, correlation, outcome fitness, and integrity across four dimensions based on the included published evidence and our own findings. This study serves as an application case of TRAEC, with 40 items of research evidence on the toxicity of MNPs to the placenta, which were rigorously screened and incorporated into the final scoring system. The final score for this TRAEC case study is 5.63, suggesting a moderate-to-low risk of reproductive toxicity associated with MNPs in the placenta, which may potentially increase with decreasing particle size. It is essential to emphasize that the findings also report original data from assays indicating that exposure to high-dose groups (100 μg/mL, 200 μg/mL) of 50 nm and 200 nm polystyrene nanoplastics (PS-NPs) induces HTR8/SVneo cell cycle arrest and cell apoptosis, which lead to reproductive toxicity in the placenta by disrupting mitochondrial function. Overall, this study employed the TRAEC strategy to provide comprehensive insight into the potential reproductive health effects of ubiquitous MNPs.

## 1. Introduction

Recently, there has been an exponential surge in both the awareness of and research on microplastics (with an approximate diameter of <5 mm) and nanoplastics (ranging from 1 to 100 nm) (MNPs) [1,2]. The escalation can be attributed to the extensive utilization of plastic products in production and daily life; there is an ongoing detection of microplastics (MPs) throughout the human body [3,4,5]. Since detecting small plastic fibers and debris in the environment is challenging, our current assessments of environmental plastic pollution may be underestimated [6]. Plastic particles are inevitably deposited into the human body through involuntary ingestion, inhalation, or skin contact [7]. Researchers worldwide have detected various types and forms of MPs in different tissues and body fluids [8], including the lungs [9], liver [10], amniotic fluid [11], blood clots [12], and placenta [13]. MPs in the environment persistently weather and degrade into finer nanoplastics (NPs), possessing smaller dimensions and larger surface areas [14]. NPs may potentially cross human biological barriers more easily compared to microplastics, leading to increased toxic accumulation [15]. A study utilizing in vitro models of intestinal Caco-2 cells and liver HepaRG cells demonstrated that 20 nm polystyrene particles elicit more pronounced effects on cell viability and apoptosis compared to particles with diameters of 40 nm and 1 μm [16]. The concept of size-dependent absorption and distribution of MNPs is consistently supported by empirical evidence. The proposed threshold of 200 nm as a research boundary for biological barriers seems ambiguous, particularly since smaller NPs have been found in excised carotid plaque specimens [17,18]. The relationship between the toxicity of plastic particles and particle size persists as a focal point of concern.

Pregnancy is a period characterized by heightened sensitivity to environmental chemicals. The first detection of MPs in the placenta in 2021 garnered significant attention [19]. While direct evidence linking MNPs to reproductive toxicity in the placenta and their impact on offspring health is currently insufficient, recent findings of microplastics in meconium [20], amniotic fluid [11], and breast milk [21] indicate that tiny plastic particles can cross the placental barrier, entering both maternal and fetal circulation. Commercialized plastic products incorporate numerous industrial compounds to enhance stability and aesthetics, while plastics exposed to ecological environments adsorb persistent organic pollutants, heavy metals, antibiotics, and other environmental contaminants through diverse molecular interactions [22,23]. Whether the adsorption strength of MNPs is related to particle size remains to be further explored. Dietary intake represents one of the primary routes of MNP exposure during pregnancy. Recent research has indicated that the particle size of MNPs influences the binding of proteins and surface charge groups on plastics. Human salivary proteases showed a stronger protein response to 50 nm particles than to 1 μm plastic particles, which led to alterations in surface group modifications. These modifications resulted in greater toxic effects upon validation across various cell lines [24]. Prenatal exposure to MNPs possibly jeopardizes fetal growth and development, potentially posing a threat to fetal abnormalities or miscarriage, which underscores a critical public health concern. However, many studies simply use MNP concentrations detected in aquatic environments to estimate exposure doses in in vivo and in vitro experiments, causing an unrealistic overestimation of particle doses [25,26]. The conversion between actual human exposure doses and experimental application doses of microplastics continues to be a substantial challenge. Given the heterogeneity in size and types of MNPs, an integrated strategy is required to consolidate evidence from various sources and to conduct a comprehensive risk assessment to evaluate adverse pregnancy outcomes caused by reproductive toxicity from these particles in the placenta.

The Targeted Risk Assessment of Environmental Chemicals (TRAEC) strategy epitomizes an innovative approach developed by our team, which proposes a standardized procedure for evaluating the risk of environmental chemical exposure. This strategy integrates evidence from various sources, including published literature and our own research findings, to perform multidimensional risk assessments of target chemicals while addressing the limitations of traditional toxicity assessment methods. Currently, relatively few studies focus on adverse pregnancy outcomes associated with MNP exposure. A systematic assessment of potential risks is facilitated by the integration of existing research evidence, thereby identifying key factors that may impact study outcomes. Meanwhile, during our evidence collection process, and given the limited number of in vitro studies on the placental toxicity of MNPs and the inconsistent conclusions obtained from these studies, we supplemented our own in vitro findings as additional evidence and combined them with published studies to provide a more comprehensive risk assessment of the reproductive toxicity of MNPs in the placenta. The theoretical risk assessment highlighted areas of concern, while the experiments provided concrete evidence to substantiate or refine these findings, resulting in more robust and defensible results. Therefore, this study utilized the TRAEC strategy to comprehensively supplement and assess MNP-induced reproductive toxicity in the placenta, providing persuasive evidence to enhance the understanding of the risks associated with prenatal exposure to MNPs.

## 2. Materials and Methods

### 2.1. Reproductive Toxicity Risk Assessment of MNPs in the Placenta Based on TRAEC Strategy

The Targeted Risk Assessment of Environmental Chemicals (TRAEC) strategy was utilized in this study to assess the reproductive toxicity in the placenta induced by MNPs. The study was conducted in accordance with the TRAEC framework, encompassing the following components. Firstly, the scientific question was proposed: “What is the correlation between MNP exposure during pregnancy and reproductive toxicity in the placenta?”, based on the following: (1) The presence of microplastics was detected in human placental tissues; (2) Relevant studies have reported that micro/nanoplastics can induce reproductive toxicity. However, their impact on the placenta remains uncertain. Subsequently, a comprehensive and systematic compilation of available evidence, including epidemiological, in vivo, and in vitro studies, is achieved through targeted evidence searches and self-conducted experiments. Finally, all the collected evidence was comprehensively assessed based on the scoring scale and risk matrix of the TRAEC strategy, with quantitatively assigned assessments in the dimensions of the reliability of study design and implementation, the correlation between exposure and health outcomes, the outcome fitness of results and integrity scores, in order to obtain strength of evidence scores and risk ratings for reproductive toxicity of MNPs in the placenta.

### 2.2. Review of Literature

Papers related to prenatal MNP exposure and the placenta were retrieved from PubMed and Web of Science up to 1 April 2024. Key search terms included (((nanoplastics) OR (microplastics) OR (plastic particles))) AND (((placenta) OR (trophoblast) OR (placental toxicity) OR (placental disorders) OR (placental insufficiency)) OR ((adverse pregnancy outcomes) OR (female reproduction) OR (female reproductive toxicity) OR (reproduction) OR (reproductive toxicity))). Duplicates from the two databases were removed for the initial screening phase. Non-compliance, reviews, and non-peer-reviewed publications were excluded from re-screening by examining the title and abstract. A careful full-text reading was finally conducted to exclude studies lacking available data, compliance, and extractable data.

### 2.3. Characterization of PS-MNPs

50 nm PS-NPs (50 mg/mL; YM1000; Yuan Biotech, Shanghai, China), 200 nm PS-NPs (2.5% [*w*/*v*]; KBsphere 0.2PS; KBsphere Tech, Suzhou, China) and 2 μm PS-MNP stock solution (2.5% [*w*/*v*]; KBsphere 2PS; KBsphere Tech) were all suspended in deionized water. The morphology of PS-MNPs was observed using the transmission electron microscope (TEM, JEM-1400Flash; JOEL) at an acceleration voltage of 120 kV and scanning electron microscope (SEM, JSM-7900F; JOEL) at an acceleration voltage of 3.0 kV. The chemical composition of 50 nm, 200 nm, and 2 μm PS-MNPs was analyzed using laser confocal Raman (LabRAM Odyssey, HORIBA France SAS, Alfortville, France), with the methodology referenced from our previous research [8,12]. Briefly, the 50× objective was used to focus on the sample surface, followed by a 532 nm laser at 10% intensity, with an acquisition time of 10 s and an acquisition number of 2 times. The spectra of the samples were generated and compared with the standard spectral library using KnowItAll software (version 23.2.50) (BioRad Laboratories, Inc., Berkeley, CA, USA). MNP identification was confirmed when the corresponding hit quality index (HQI) exceeded 70.

### 2.4. Cell Culture and Treatment

HTR-8/SVneo (ZQ0482; Zhong Qiao Xin Zhou, Shanghai, China) were cultured in RPMI 1640 medium (PM150110; Procell, Wuhan, China) supplemented with 10% fetal bovine serum (164210-50; Procell, Wuhan, China) and antibiotics (100 U/mL penicillin and 100 μg/mL streptomycin) at a temperature of 37 °C in a humidified incubator containing 5% CO_2_. During exposure assays, the PS-MNPs suspended in deionized water were sonicated for 20 s to fully mix and distribute evenly. The PS-MNP suspension was subsequently diluted with culture medium and thoroughly mixed using a vortex mixer before being added to cell culture dishes or plates. HTR-8/SVneo cells were subjected to fresh medium containing PS-MNP particles (PS 50 nm, 200 nm, and 2 μm) at various concentrations of 0, 10, 50, 100, and 200 μg/mL, respectively, and the culture medium was gently mixed periodically.

### 2.5. Cell Counting Kit-8 (CCK-8) Assay

The HTR-8/SVneo cells were seeded in 96-well plates at a density of 1 × 10^4^ cells/well and incubated for 12 h. Subsequently, the original culture medium was replaced with 100 μL of medium containing varying concentrations of PS-MNPs (0, 10, 50, 100, and 200 μg/mL) for additional culture durations of 0 h, 12 h, 24 h, and 48 h. Three replicate wells were established for each experimental group. CCK-8 reagent (A311-01; Vazyme, Nanjing, China) was added to each well at different time points during the assay. After incubation for 2 h in a light-free environment, the cellular activity was assessed by measuring the absorbance at 450 nm using the microplate reader (InfiniteM 200Pro; Tecan, Männedorf, Switzerland).

### 2.6. Wound Healing Assay

HTR-8/SVneo cells were seeded in 6-well plates at a density of 6 × 10^5^ cells/well and then cultured in a constant temperature incubator. When the cell confluence reached 90% or more, a vertical wound was made in the center of each well with the tip of a sterile 200 μL pipette gun. After washing twice with PBS, serum-free medium containing 0, 10, 50, 100, and 200 μg/mL PS-MNPs was added. The cells were cultured in a constant temperature incubator, and the representative images of scratch profiles were observed under an inverted microscope (Eclipse Ti; Nikon Corporation, Tokyo, Japan) at 0 h, 12 h, and 24 h. The measurement and analysis of cell migration were conducted using the ImageJ software (version 1.45j). The mathematical formula is as follows: migration rate (%) = (A_0_ − A_t_)/A_0_ × 100%. The initial wound area (A_0_) was measured at 0 h, while the subsequent wound areas (A_t_) were measured either at 12 h or 24 h.

### 2.7. Transwell Migration Assay

Transwell chambers (3422; Corning, Corning, NY, USA) with an 8 μm pore size were inserted into 24-well plates, and a layer of matrigel was applied to the chambers. HTR-8/SVneo cells were seeded in the upper chambers with serum-free medium at a density of 1 × 10^5^ cells/well, while the lower chamber was supplemented with medium containing PS-MNPs at concentrations of 0, 10, 50, 100, and 200 μg/mL prepared with medium supplemented with 20% fetal bovine serum. After a 24-h incubation period, HTR-8/SVneo cells were fixed using the 4% paraformaldehyde solution (BL1280A; Biosharp, Hefei, China) for 30 min, followed by PBS washing and subsequent staining with 0.1% crystal violet staining solution (BL802A; Biosharp, Hefei, China) for another 30 min. Afterward, the cells were gently removed from the chamber sidewalls utilizing sterile cotton swabs, and images were captured utilizing an inverted microscope (Eclipse Ti; Nikon Corporation, Tokyo, Japan).

### 2.8. Cell Cycle Analysis

HTR-8/SVneo cells were seeded in 6 cm cell culture dishes and treated with 0, 10, 50, 100, 200 μg/mL of PS-MNPs. Upon reaching 80% cell confluence, cells were collected and gently mixed with pre-cooled 70% ethanol and fixed at 4 °C for 18 h or more. After fixation, the precipitated suspension cells were resuspended in 300 μL of PI/RNase staining buffer (550825; BD Biosciences, Franklin Lakes, NJ, USA). After incubation at 37 °C for 15 min in the absence of light, the sample was filtered using a membrane filter and detected via LSRFortessa flow cytometer (BD Biosciences, Franklin Lakes, NJ, USA).

### 2.9. Cell Apoptosis Detection

HTR-8/SVneo cells were seeded at a density of 6 × 10^5^ cells/well in 6-well plates and exposed to 0, 10, 50, 100, and 200 μg/mL PS-MNPs for 24 h. The cells were collected with an EDTA-free trypsin digestion solution (C0205; Beyotime, Shanghai, China), and the suspended cell precipitates were added with 300 μL Binding Buffer for each sample, and then 5 μL FITC AnnexinV and 5 μL Propyl Iodide (PI) staining solution (556547; BD Biosciences, Franklin Lakes, NJ, USA) were added to homogenously mix the suspended cells. The cells are kept in the dark and incubated at room temperature for 15 min, then filtered through a filter membrane and analyzed using an LSRFortess flow cytometer (BD Biosciences, Franklin Lakes, NJ, USA).

### 2.10. Mitochondrial Membrane Potential (MMP) Assay

HTR-8/SVneo cells were seeded in 6-well plates at a density of 6 × 10^5^ cells/well and exposed to 0, 10, 50, 100, and 200 μg/mL PS-MNPs for 24 h. Cells were subjected to treatment with CCCP at a concentration of 1:1000 for a duration of 20 min, serving as the positive control group. The Mitochondrial membrane potential (MMP) of cells was detected using the Mitochondrial membrane potential assay kit with JC-1 (C2006; Beyotime, Shanghai, China). Following cell collection, the cells were incubated with JC-1 staining working solution at 37 °C in the cell culture incubator for 20 min. The cells were subsequently washed twice with 1x JC-1 dye buffer and filtered through a membrane filter. The fluorescence intensities were analyzed using an LSRFortess flow cytometer (BD Bioscience, Franklin Lakes, NJ, USA).

### 2.11. Statistical Analysis

The statistical analysis was performed using GraphPad Prism software (version 10.0). A two-tailed Student’s *t* test was used to compare the differences between the two groups, and a one-way analysis of variance (ANOVA) was used to compare the differences among multiple groups. All data were presented as mean ± standard error of mean, and statistical significance was defined as *p* < 0.05.

## 3. Results

### 3.1. Evidence Search and Selection of Prenatal Exposure to MNPs

**Figure 1:**
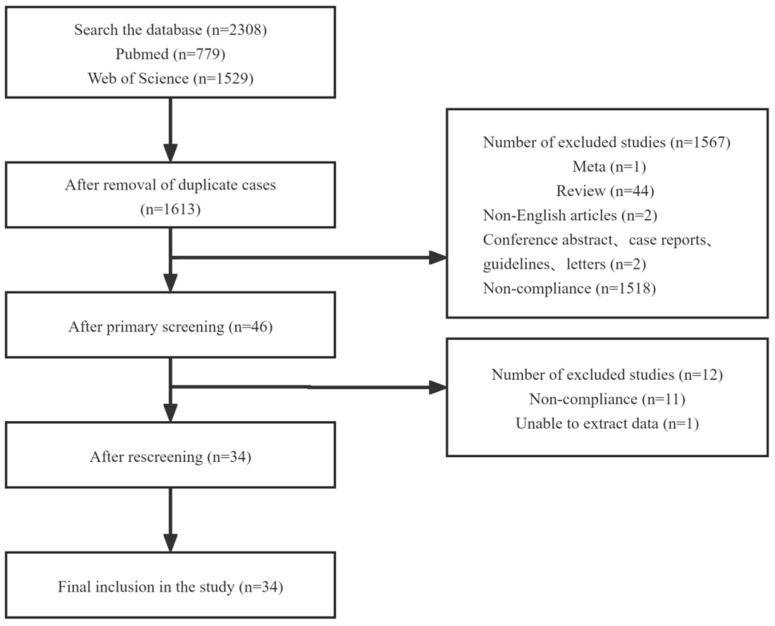
Article review flow diagram.

To acquire a relatively complete overview of current research findings on the effect of prenatal MNP exposure on the placenta, a literature survey was conducted to review all peer-reviewed journal articles. The detailed screening process and publication counts are depicted in Figure 1, and finally, 34 relevant articles, including 39 items of research evidence, were selected for the evaluation of the TRAEC strategy in this study, comprising 13 epidemiological studies (Table 1), 14 in vivo studies (Table 2), and 12 in vitro studies (Table 3).

### 3.2. Overview of the Potential Effects of MNPs on the Placenta

The placenta serves as a vital intermediary organ, maintaining a healthy pregnancy by facilitating the exchange of materials, defense mechanisms, immunity, and hormone synthesis between the mother and fetus [27,28]. Ragusa et al. reported the initial identification of synthetic particles and pigment MPs on both the maternal and fetal aspects of the placenta, sparking concerns about maternal–fetal exposure to MNPs and associated substances at this interface [19]. Subsequent population-based studies found an increased quantity and variety of plastic particles in the placenta [29], amniotic fluid [11], meconium [20], and breast milk [21] samples. However, direct evidence confirming the association between microplastics and adverse pregnancy outcomes remains scarce. A study in 2024 collected villous samples from both recurrent miscarriage (RM) patients and healthy control (HC) pregnant women who opted for elective abortion. It revealed elevated levels of PS particles in the RM group in contrast to the HC group. In addition, through a mouse pregnancy exposure model, the study confirmed that high doses of PS-NPs induced abortion by promoting placental apoptosis [30]. This underscores plastic particle exposure as an emergent risk factor for unexplained recurrent miscarriage.

Most in vivo studies utilize murine models to simulate prenatal exposure to MNPs and investigate their impacts on placental or offspring development. Following late gestational inhalation of MPs, rats exhibited particle deposition in fetal tissues postnatally, which were transferred from maternal uterine circulation through the placenta into fetal circulation [31]. Additionally, mouse prenatal exposure to MNPs was associated with elevated placental dysfunction and fetal hypoxia hemodynamic markers, with NPs exerting a more pronounced impact on murine placental function compared to MPs [32]. The ex vivo placental perfusion model was employed to delve into particle transport mechanisms, revealing a transcriptional induction of inflammatory responses in trophoblast cells triggered by MP exposure [33]. Further in vitro experiments demonstrated a dose-dependent generation of pro-inflammatory cytokines IFN-γ and TNF-α upon the MP’s entry into trophoblast cells [34]. Another study explored the toxicity of varying sizes of MPs, indicating that smaller particles induce greater toxicity by causing trophoblast cell cycle arrest through increased ROS production [35]. To discern toxicity nuances between micro-, submicro-, and nanoparticles, human trophoblast cells were exposed to PS-MNPs sized at 2 μm, 200 nm, and 50 nm to observe alterations in cellular function.

**Table 1:** Epidemiologic study to assess placental MNP exposure.

Number	Experimental Subject	Quantity and Type	Research Results	Conclusion	Literature
1	6 placentas	Twelve microplastic fragments, ranging from 5 to 10 μm and exhibiting spheric or irregular shapes found in four placentas. The distribution of these fragments was as follows: five on the fetal side, four on the maternal side, and three in the chorioamniotic membranes.	Several microplastic fragments were detected in human placenta samples from six pregnant women with uneventful pregnancies using Raman microscopic spectroscopy.	+	Ragusa et al. (2021) [19]
2	18 placentas and 12 meconium	Sixteen types of MPs were detected in the placentas and meconium, with polyamide (PA) and polyurethane (PU) being the main types. The detection rate of MPs in samples with particle sizes ranging from 20 to 50 μm was all above 76.46%.	A variety of microplastics were found in placentas and meconium samples, some of which may be related to the microbiota genera in placentas and meconium.	+	Liu et al. (2023) [21]
3	18 placentas and 18 meconium	The detection revealed the presence of 16 types of MPs across all samples, with polyamide (PA) and PU accounting for over 78% of the total MP particles. The median concentration of MPs detected in placental samples was 18.0 particles/g, with more than 76.46% of the MP particles within the size range of 20~50 μm.	MPs are ubiquitous in placentas and meconium samples, and exposure to several MPs was associated with microbiota in placentas and meconium.	+	Liu et al. (2023) [20]
4	60 Villous tissues from 30 patients with unexplained miscarriage (UM group) and 30 women with elective miscarriage (HC group)	PS fragments were detected in human villous tissues, and the content of PS fragments in UM villi tissue was higher than that in HC villous tissues.	The contents of PS plastic fragments might have a strong predictive ability for miscarriage, with high specificity and sensitivity.	+	Wan et al. (2024) [36]
5	17 placentas	The MPs were primarily composed of polyvinyl chloride, polypropylene, and polybutadiene. The particle sizes of these microplastics ranged from 20.34 to 307.29 μm, with the majority (80.29%) being smaller than 100 μm.	More microplastics were detected using the LD-IR technique compared to previous studies, which suggests that more microplastics might accumulate in the placenta than previously estimated.	+	Zhu et al. (2023) [29]
6	12 placentas	By using UHPLC coupled with mass spectrometry, 36 samples of polyethylene glycol (PEG) compounds were detected in 12 human placenta samples.	Upon entering the human body, PEG can reach all levels of placental tissues and potentially modify the connection between mother and fetus.	+	Ragusa et al. (2022) [37]
7	10 placentas	MP-compatible particles were present on the surface of the placental villi, in the intracellular environment of different placental cell layers, or within the extracellular environment.	MP-compatible particles exist on the surface of the placental villi, within the intracellular environment of various placental cell layers, or in the extracellular environment.	+	Ragusa et al. (2022) [38]
8	62 placentas	The Py-GC-MS results showed the presence of microplastics in the placentas of all participants, with concentrations ranging from 6.5 to 685 μg NMPs per gram of placental tissue, averaging 126.8 ± 147.5 µg/g (mean ± SD). Polyethylene was the most common polymer.	NMPs have the propensity to accumulate in placentas at significantly higher concentrations compared to those detected in blood samples.	+	Garcia et al. (2024) [13]
9	36 Villous tissues from 18 patients with unexplained miscarriage (UM group) and 18 women with elective miscarriage (HC group)	PS fragments were detected in villous tissues from patients with recurrent abortion (RM) and healthy control (HC) patients (18 cases each). The content of PS fragments in the HC group ranged from 0 to 1.68 mg/kg, and that in the RM group ranged from 0.56 to 4.13 mg/kg.	PS plastic fragments were present in human villous tissues, and the contents of PS plastic fragments were higher in the RM group than in the HC group. Additionally, there is a positive association between the contents of PS plastic fragments in villous tissues and miscarriage.	+	Wan et al. (2024) [30]
10	30 placentas	Raman spectroscopy analysis revealed the presence of MP particles in 6 out of 10 placentas in 2006, 9 out of 10 placentas in 2013, and all examined placentas in 2021. All identified MP particles were similar in size range and color.	Over the past 15 years, there has been a significant increase in the accumulation of microplastics in waste placentas, and both the size and chemical composition of the polymers have also changed.	+	Weingrill et al. (2023) [39]
11	43 placentas	MPs were found in 13 intrauterine growth restriction (IUGR) pregnancies, with an average abundance ranging from 2 to 38 particles per placenta. However, in normal pregnancies, MPs were below the limit of detection (LOD) except for three out of the 30 subjects.	A negative association was observed between exposure to MPs and adverse neonatal anthropometric measurements in pregnant women with IUGR pregnancies compared to those without IUGR.	+	Amereh et al. (2022) [40]
12	3 placentas, 2 meconium and 2 maternal stools	Polyethylene, polypropylene, polystyrene, and polyurethane were detected in the placenta and meconium samples.	MPs > 50 µm have been found in placenta and meconium samples obtained from cesarean delivery. Critical evaluation of potential contamination sources is crucial, and this may help guide future clinical research to improve the correct detection of MPs in organ tissue.	+	Braun et al. (2021) [41]
13	10 placentas and 10 amniotic fluids	Forty-four microplastic particles and polymer additives were identified in all samples. The particle size of chlorinated polyethylene (CPE) and calcium-zinc PVC stabilizers were between 10 and 50 μm.	Evidence has been provided indicating the presence of microplastics and additives in samples of human amniotic fluid and placenta, thus enhancing the understanding of the potential transmission of microplastics and additives from the mother’s blood to the fetus through the placenta and amniotic fluid.	+	Halfar et al. (2023) [11]

**Table 2:** In vivo studies of the placenta effects of MNPs in mammals.

Number	Experimental Subject	Exposure Mode	Research Results	Conclusion	Literature
1	C57BL/6-mated BALB/c mice	At days 5.5 and 7.5 of gestation, 10 μm PS-MP particles were injected into a saline solution of 200 μL at a dose of 250 μg.	In an allogeneic mating murine model, PS-MP exposure resulted in fetal losses due to disruption of the immune microenvironment.	+	Hu et al. (2021) [42]
2	C57BL mice	1 μm PS MPs (1 mg/day), 100 nm PS NPs (1 mg/day), PS NPs-COOH (1 mg/day) or combined PS particles (NPs 1 mg/day and MPs 1 mg/day, or NPs-COOH 1 mg/day and MPs 1 mg/day) was conducted by intragastric administration for 17 consecutive days.	The injury of MPs to placental tissue promoted the entrance of nanoparticles into BPB.	+	Yang et al. (2022) [35]
3	C57 BL/6 mice	1 and 10 mg/L 100 nm PS-NP were exposed via drinking water for 17 days.	PS-NPs resulted in fetal growth restriction and significantly disrupted cholesterol metabolism in both the placenta and fetus.	+	Chen et al. (2023) [43]
4	Sprague Dawley rats	At the 19th day of gestation, 20 nm nanopolystyrene beads labeled with Rhodamine were injected into the trachea for 24 h.	Exposure to nanopolystyrene in the maternal lungs leads to the translocation of plastic particles to both the placenta and fetal tissues, rendering fetal placental unit susceptible to adverse effects.	+	Fournier et al. (2020) [44]
5	C57BL/6 mice	100 mg/kg 50 nm PS-NPs or an equal amount of saline was administered via oral gavage from D1 to D13.	Exposure to PS-NP could lead to miscarriage in pregnant mice. Mechanistically, PS-NPs activated autophagy inhibited SOX2-mediated ROCK1 transcription and suppressed Rock1-mediated migration/invasion and the formation of migrasomes, ultimately resulting in miscarriage.	+	Wan et al. (2024) [36]
6	CD-1 mice	At four different concentrations (0 ng/L (control group), 10^2^ ng/L, 10^4^ ng/L, 10^6^ ng/L), the filtered water contained 5 μm polystyrene microplastics throughout pregnancy.	Maternal exposure to microplastics led to significant alterations in placental metabolism.	+	Aghaei et al. (2022) [45]
7	C57BL/6 mice	From D1 to D14, 50 nm PS-NPs were orally administered via gavage at doses of 0.25, 0.5, 1 or 2 mg/kg to mice.	Exposure to PS-NPs triggered the activation of Bcl-2/Cleaved-caspase-2/Cleaved-caspase-3, resulting in excessive apoptosis in the mice’s placental tissues and subsequently inducing miscarriage.	+	Wan et al. (2024) [30]
8	CD-1 mice	10^6^ ng/L polyethylene microplastics and nanoplastics, with a size range of 740 to 4990 nanometers (dissolved in 0.1% surfactant solution, filtered drinking water).	Compared with the control group, the umbilical artery blood flow increased by 43% in the polyethylene group, which had a significant impact on placental function.	+	Hanrahan et al. (2024) [46]
9	ICR mice	At E8.5, E9.5, or E10.5, 300 lg of 60 nm or 900 nm PS-NPs or saline (control) were injected intravenously into the mice; At E15, 60 nm or 900 nm PS-NPs or 300 mL of saline were injected again.	60 nm or 900 nm PS-NPs could penetrate the placenta of mice and affect the developing mice fetuses.	+	Nie et al. (2021) [47]
10	Mice	An equivalent amount of suspensions containing 50–70 nm carboxylated or PEG-modified nanoparticles was administered via the tail vein at a dose of 7 μL/g of body weight for either 5 min or 4 days.	The distribution of carboxylated or PEG-conjugated polystyrene nanoparticles in the body was analyzed, and the results showed that the placenta has an appropriate barrier function. When NPs were trapped in the lacunas of the placenta, barriers completely prevented the particles from entering the fetal tissues.	+	Kenesei et al. (2016) [48]
11	Sprague Dawley rats	Sprague Dawley rats were given 10 mL/kg of 25 nm carboxylated polystyrene spheres with a concentration of 250 µg/mL on the 19th day of gestation and were sacrificed 24 h later.	The nanosized polystyrene MNPs ingested can disrupt the maternal–fetal barrier of the placenta.	+	Cary et al. (2023) [49]
12	CD-1 mice	5 μm PS-MP or 50 nm PS-NPs were dissolved in drinking water at a concentration of 10^6^ ng/L.	Maternal exposure to PS-MPs and PS-NPs resulted in abnormal placental blood flow, the extent of which depended on the size of the plastic particles.	+	Dibbon et al. (2024) [32]
13	CD-1 mice	Polystyrene plastics with a size of 5 μm or 50 nm were dissolved at concentrations of 10^2^, 10^4^, or 10^6^ ng/L in filtered drinking water.	The growth of fetuses exposed to MP and NP is significantly restricted in late gestation, with a 12% reduction in fetal weight at the highest exposure concentration.	+	Halfar et al. (2023) [11]
14	Sprague Dawley rats	4.34 × 10^14^ 20 nm rhodamine-labeled polystyrene beads	Maternal exposure to NPs resulted in widespread functional changes in the placental circulation.	+	Cary et al. (2024) [31]

**Table 3:** In vitro studies of the placenta effects of MNPs in mammals.

Number	Experimental Subject	Exposure Mode	Research Results	Conclusion	Literature
1	BeWo b30 choriocarcinoma cell line (nonsyncytialized and syncytialized cells)	Exposed to fluorescent and nonfluorescent PS (0.05, 0.2, 1, and 10 μm) and High-density polyethylene (0–80 μm) at 0.1, 1, 10, and 100 μg/mL for 24 h	Pristine and weathered MNPs are internalized and translocated by placental cells in vitro.	+	Dusza et al. (2022) [50]
2	Human choriocarcinoma HLA-G-positive cell line (JEG-3)	100 nm NPs, 100 nm NPs-COOH, or 1000 nm MPs at concentrations of 0, 60, 120, 240, and 480 μg/mL for 24 h or 48 h	Exposure to micro and/or nanoparticles resulted in decreased proliferation of JEG-3 cells, as well as increased apoptosis and ROS levels.	+	Yang et al. (2022) [35]
3	Swan 71 cells	0, 50, 100, 150, or 200 μg/mL of 50 nm PS-NPs	Exposure to PS-NP could lead to miscarriage in pregnant mice. Mechanistically, PS-NPs activated autophagy inhibited SOX2-mediated ROCK1 transcription and suppressed Rock1-mediated migration/invasion and the formation of migrasomes, ultimately resulting in miscarriage.	+	Wan et al. (2024) [36]
4	Human placental choriocarcinoma (JEG-3) cells	Exposed to 25, 50, 100, 500 nm polystyrene nanoplastics with -NH2, -COOH, and unlabeled surface charges at 20, 78, 313, 1250, and 5000 μg/mL for 24 h.	PS-NPs exhibit a toxic pattern with size and surface charge specificity. The smaller PS-NP is, the greater its toxicity to human placental cells.	+	Shen et al. (2022) [51]
5	Human trophoblast HTR-8/Svneo cells	10, 50, or 100 μg/mL 100 nm PS-NPs	NPs have adverse consequences on the biological functions of trophoblasts.	+	Hu et al. (2022) [34]
6	Ex vivo placental perfusion, BeWo b30 choriocarcinoma cell line	6 h of perfusion with sub-cytotoxic concentrations of PS NPs (70 nm, 25 µg/mL) Exposed for 24 h to PS NPs (70 nm) at concentrations of up to 100 µg/mL	Exposure of maternal to CuO NP and PS nanoplastics in an ex vivo human placenta model can induce material-specific transcriptional changes in the placental tissue.	+	Chortarea et al. (2023) [33]
7	Swan 71 cells	Exposed to 0, 50, 100, 150, or 200 μg/mL of 50 nm FITC-PS-NPs	Exposure to PS-NPs at concentrations of 50, 100, 150, or 200 µg/mL activated the Bcl-2/Cleaved-caspase-2/Cleaved-caspase-3 signaling through the mitochondrial pathway, leading to increased oxidative stress in human trophoblast cells, a decrease in mitochondrial membrane potential and an increase in cell apoptosis.	+	Wan et al. (2024) [30]
8	HDN combined with a 2D placental–trophoblast model (BeWo b30) and 3D EBs	Exposed to carboxyl-modified GFP-labeled polystyrene particles of 500 nm at 0, 1, 10, or 100 μg/mL	The cellular uptake and intracellular accumulation of PS-MPs were evident in placental tissues.	+	Boos et al. (2021) [52]
9	HPEC-A2 cells and human placental choriocarcinoma cell line BeWo b30	Exposed to 50 nm PS-nano and 500 nm PS-micro for 24 h in a concentration range from 0.1 to 100 μg/mL.	Polystyrene particles at the nano and micron scales were not acutely toxic. No evidence of transport across the intestinal and placental barriers was found, but cell uptake and intracellular accumulation of PS nanoparticles and microspheres were confirmed.	-	Hesler et al. (2019) [53]
10	Co-culture transfer model with tight layers of trophoblasts (BeWo b30) and placental microvascular ECs (HPEC-A2)	Exposed to 49 nm PS NPs at 0.5 mg/mL and 70 nm PS NPs at 50 µg/mL	No translocation of 70 nm PS NPs across the placental barrier in vitro was observed, while small amounts of 49 nm PS NPs were detected in the basolateral compartment.	-	Aengenheister et al. (2018) [54]
11	Ex vivo dual recirculating human placental perfusion model	Exposed to 50, 80, 240, or 500 nm PS beads at a final concentration of 25 μg/mL	The placenta was capable of internalizing fluorescent polystyrene particles with diameters up to 240 nm, allowing them to traverse the placental barrier without affecting the viability of placental explants.	+	Wick et al. (2010) [55]
12	Ex vivo human placental perfusion model BeWo cells	Exposed to 50 and 240 nm plain (without functionalization) yellow-green–labeled PS beads at a concentration of 25 μg/mL	PS particles did not significantly reduce cell viability. The reverse perfusions (F→M direction) led to an augmented translocation of PS beads, with an accumulation of polystyrene particles within the syncytiotrophoblast layer of the placental tissue.	-	Grafmueller et al. (2015) [56]

### 3.3. The Internalization of PS-MNPs Reduces Viability of Trophoblast Cells

**Figure 2:**
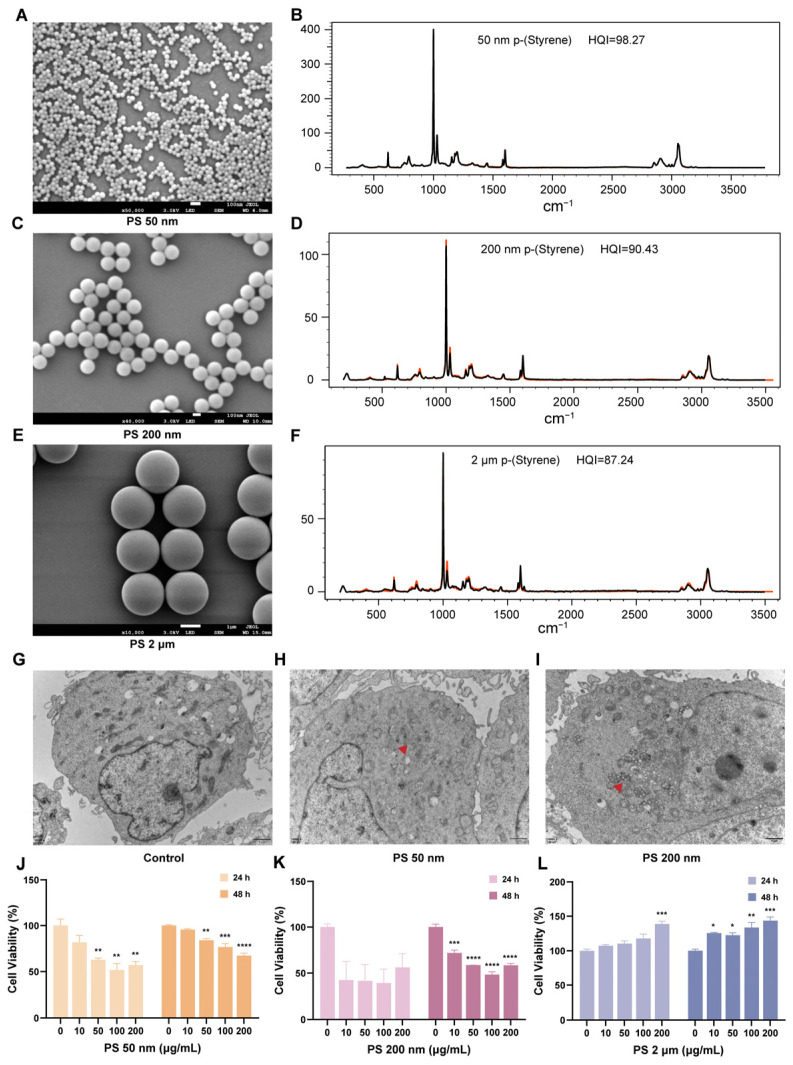
Exposure to PS-MNPs hampers the proliferation of HTR-8/SVneo cells. (**A**,**C**,**E**) The morphology of 50 nm (**A**), 200 nm (**C**), and 2 μm (**E**) PS-MNPs under SEM at an acceleration voltage of 3.0 kV; 50 nm and 200 nm PS-NPs were marked with a red arrow; (**B**,**D**,**F**) The chemical composition of 50 nm (**B**), 200 nm (**D**), and 2 μm (**F**) PS-MNPs were analyzed using laser confocal Raman. Representative spectra PS-MNPs detected. The acquired spectra were shown in black, with the greatly matched reference spectra in red; (**G**–**I**) TEM image of HTR-8/SVneo cells (Control; (**G**)) and after 48-h treatment with 100 μg/mL PS-NPs of 50 nm (**H**) and 200 nm (**I**) particles; (**J**–**L**) The proliferation capacity of HTR-8/SVneo cells was evaluated with CCK-8 after exposure to varying gradient concentrations (0, 10, 50, 100, 200 μg/mL) of PS-MNPs with sizes of 50 nm (**J**), 200 nm (**K**), and 2 μm (**L**) for durations of 24 h and 48 h. Results were presented as means ± SEM (n = 3/group). Statistical comparisons were performed using one-way ANOVA with the Tukey post hoc test. * *p* < 0.05, ** *p* < 0.01, *** *p* < 0.001, **** *p* < 0.0001 in comparison to the control group (0 μg/mL). Note: ANOVA, analysis of variance; CCK-8, cell counting kit-8; PS-MNPs, polystyrene micro/nanoplastics; PS-NPs, polystyrene nanoparticles; SEM, scanning electron microscopy; SEM, standard error of mean; TEM, transmission electron microscope.

During early pregnancy, trophoblast cells gradually differentiate into villi, later forming the placenta and membranes. Trophoblast cells function normally to maintain a healthy pregnancy state. Here, we selected human trophoblast cells (HTR-8/SVneo cells) to explore the impact of different sizes of PS-MNPs on cell function during pregnancy. Initially, we characterized particles under scanning electron microscopy (SEM) and observed that all three sizes exhibited uniform spherical shapes (Figure 2A,C,E). Laser confocal Raman spectroscopy was utilized to analyze the chemical compositions of the particles of the experimentally applied MNPs. It was shown that the plastic particles measuring 50 nm, 200 nm, and 2 μm closely conformed to the standard profiles of polystyrene, with HQI exceeding 70 (Figure 2B,D,F). After 48 h of exposure to 100 μg/mL of PS-MNPs, transmission electron microscopy (TEM) revealed internalization of 50 nm and 200 nm plastic particles within the nuclei and cytoplasm of HTR-8/SVneo cells, whereas no internalization of 2 μm PS-MPs was observed. This suggests that NPs are more prone to cellular uptake compared to MPs (Figure 2G,H and Figure S1). Cell viability assays were conducted for the three sizes of PS-MNPs at 0, 10, 50, 100, and 200 μg/mL, showing a significant dose-dependent decrease after 24 and 48 h of exposure to 50 nm PS-NPs (** *p* < 0.01, *** *p* < 0.001, **** *p* < 0.0001; Figure 2J and Figure S2A), and the exposure of 200 nm PS-NPs for 48 h affected the cell viability at a concentrations of 10, 50, 100, 200 μg/mL (*** *p* < 0.001, **** *p* < 0.0001; Figure 2K and Figure S2B). While 2 μm PS-MPs had no inhibitory effect on trophoblast cell viability, the exposure of PS-MPs at 0, 10, 50, 100, and 200 μg/mL could promote the cell activity of HTR-8 cells (Figure 2L and Figure S2C).

### 3.4. PS-NPs Reduce Trophoblast Cells Migration and Invasion Capability

**Figure 3:**
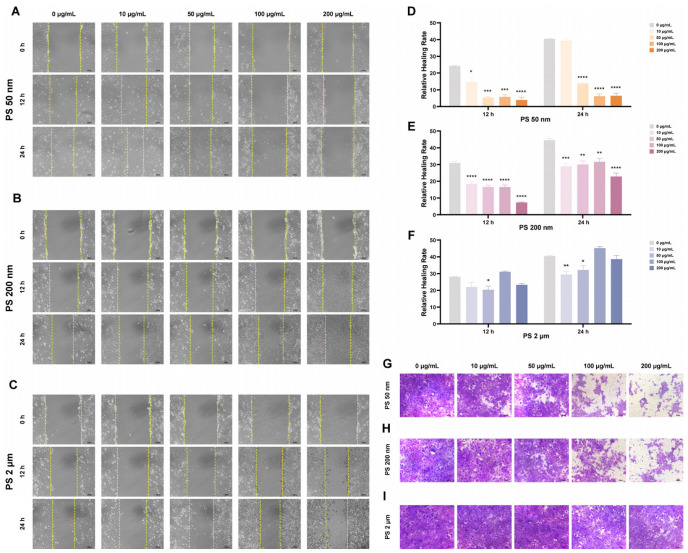
PS-MNP exposure hinders the migration and invasion of HTR-8/SVneo cells. (**A**–**F**) The impact of PS-MNPs at different concentrations (0, 10, 50, 100, and 200 μg/mL) at sizes of 50 nm (**A**,**D**), 200 nm (**B**,**E**), and 2 μm (**C**,**F**) on cell migration was evaluated by wound healing assay under a magnification of 100×. Data are represented as means ± SEM (n = 3/group). Statistical comparisons were performed using one-way ANOVA with the Tukey post hoc test. * *p* < 0.05, ** *p* < 0.01, *** *p* < 0.001, **** *p* < 0.0001 in comparison to the control group (0 μg/mL). (**G**–**I**) Representative images of HTR-8/SVneo cells were captured after exposure to PS-MNPs at concentrations of 0, 10, 50, 100, and 200 μg/mL with sizes of 50 nm (**G**), 200 nm (**H**), and 2 μm (**I**) for a duration of 24 h by Transwell assays under a magnification of 200×. Notes: ANOVA, analysis of variance; PS-MNPs, polystyrene micro/nanoplastics; SEM, standard error of mean.

Wound healing assays were performed on HTR-8/SVneo cells exposed to PS-MNPs for 12 h and 24 h of 50 nm, 200 nm, and 2 μm at concentrations of 0, 10, 50, 100, and 200 μg/mL (Figure 3A–C). It was found that 50 nm and 200 nm PS-NPs significantly impacted the migration and invasion ability in a manner dependent on both time and dose. Specifically, compared with the control and low-dose groups (0, 10, 50 μg/mL), the high-dose groups (100, 200 μg/mL) significantly affected the migration ability of cells under the condition of 24 h exposure duration (* *p* < 0.05, ** *p* < 0.01, *** *p* < 0.001, **** *p* < 0.0001; Figure 3D,E). Staining of invasive cells with crystal violet revealed that 50 nm and 200 nm PS-NPs similarly reduced the number of infiltrating cells after 24 h of exposure at high-dose groups (100, 200 μg/mL) (Figure 3G,H). Under the same concentration and time conditions, no significant difference in the trophoblast cell migration rate or the degree of cell invasion was observed between the high-dose groups (2 μm PS-MPs) and the control group (Figure 3F,I).

### 3.5. PS-NPs Induce Trophoblast Cell Cycle Arrest and Increase Apoptosis

**Figure 4:**
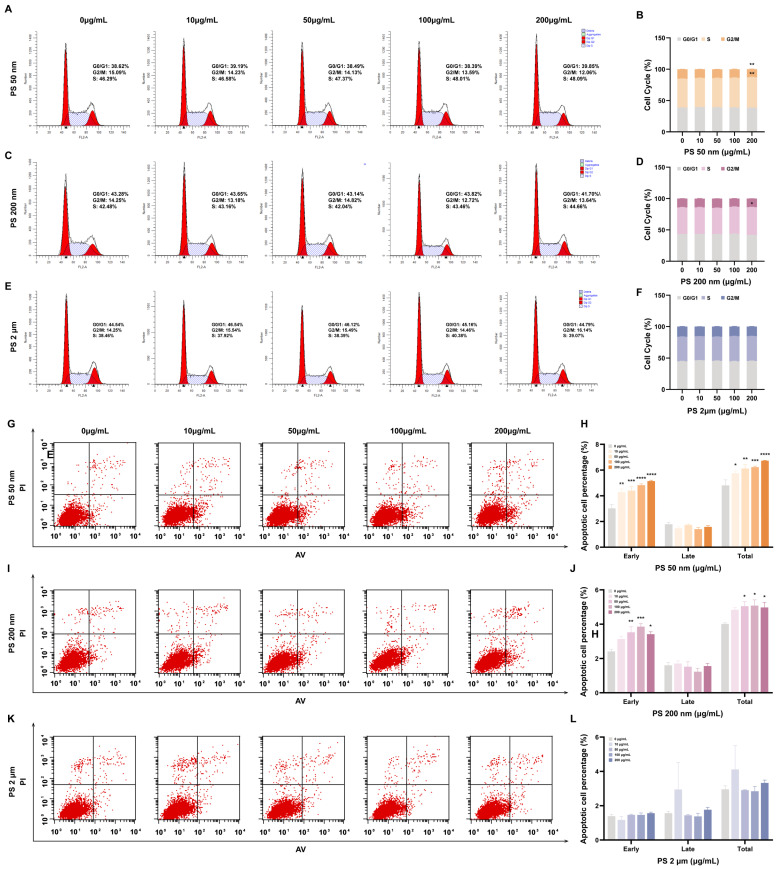
Exposure to PS-MNPs can impede the cell cycle progression and trigger cellular apoptosis. (**A**–**F**) Cell cycle assay was conducted after exposure to different sizes of 50 nm (**A**,**B**), 200 nm (**C**,**D**), 2 μm (**E**,**F**) with concentrations of 0, 10, 50, 100, 200 μg/mL of PS-MNPs for 48 h by flow cytometer. Data were analyzed using FlowJo software (version 10.10) and presented as means ± SEM (n = 3/group). Statistical analyses were performed using one-way ANOVA followed by the Tukey post hoc test. * *p* < 0.05, ** *p* < 0.01 in comparison to the control group (0 μg/mL); (**G**–**L**) Flow cytometry was used to obtain results from the apoptosis assay conducted on HTR-8/SVneo cells treated with 50 nm (**G**,**H**), 200 nm (**I**,**J**), 2 μm (**K**,**L**) PS-MNPs for 48 h, encompassing doses ranging from 10 to 200 μg/mL. Data were analyzed using FlowJo software and presented as means ± SEM (n = 3/group). Statistical comparisons were conducted using one-way ANOVA with the Tukey post hoc test. * *p* < 0.05, ** *p* < 0.01, *** *p* < 0.001, **** *p* < 0.0001 in comparison to the control group (0 μg/mL). Notes: ANOVA, analysis of variance; PS-MNPs, polystyrene micro/nanoplastics; SEM, standard error of mean.

HTR8/SVneo cells exposed to the highest dose of 50 nm PS-NPs (200 μg/mL) increased the S-phase of cells from 46.29% to 48.09% (** *p* < 0.01) and decreased the G2/M-phase from 15.09% to 12.06% (** *p* < 0.01) compared to the control group (Figure 4A,B). The S phase of cells exposed to 200 nm PS-NPs increased from 42.48% to 44.66% (* *p* < 0.05) compared to the control group (Figure 4C,D). This suggests that high doses of 50 nm and 200 nm PS-NPs significantly affected trophoblast cell cycle progression, resulting in increased cell accumulation in the S phase and blocking of the DNA replication process. Upon further examination with Annexin V and Propidium Iodide staining to detect apoptosis, a notable increase in early apoptosis was observed in cells exposed to 200 nm and 50 nm plastic particles (* *p* < 0.05, ** *p* < 0.01, *** *p* < 0.001, **** *p* < 0.0001; Figure 4G–J). We inferred that this phenomenon stems from cell cycle arrest at the G0/G1 phase, impeding DNA replication in some cells and consequently inducing apoptosis. HTR8/SVneo cells exposed to 2 μm PS-MPs displayed normal cell cycle progression (Figure 4E,F), with no significant difference in apoptotic cell count (Figure 4K–L).

### 3.6. PS-NPs Decrease Mitochondrial Membrane Potential in Trophoblast Cells

**Figure 5:**
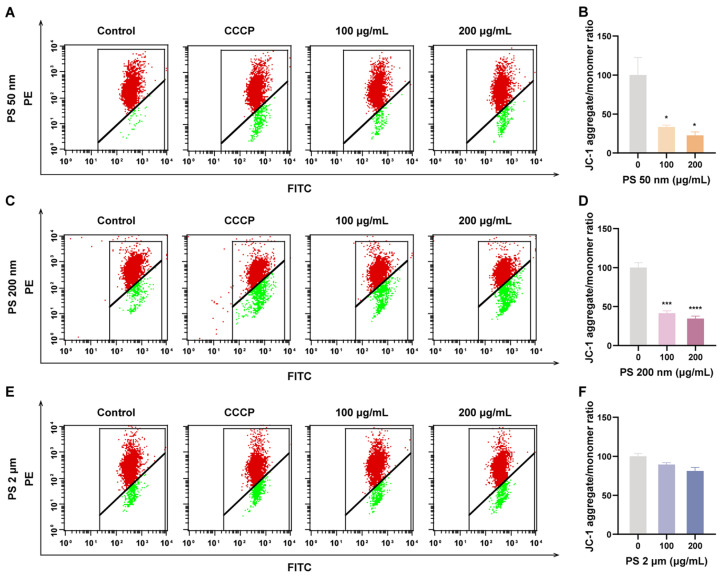
Exposing HTR-8/SVneo cells to PS-MNPs resulted in mitochondrial depolarization, leading to mitochondrial damage. (**A**,**B**) MMP (ΔΨm) of trophoblast cells was assessed by flow cytometry after staining with JC-1 after exposure to 50 nm PS-NPs at concentrations of 0 (Control), 100, 200 μg/mL for 48 h; (**C**,**D**) JC-1 Dye analysis of MMP (ΔΨm) was performed by flow cytometry. The cells were exposed to 0 (Control), 100, and 200 μg/mL of 200 nm PS-NPs for 48 h; (**E**,**F**) The MMP (ΔΨm) of HTR-8/SVneo cells treated with 2 μm PS-MPs at concentrations of 0 (Control), 100, and 200 μg/mL were assessed using JC-1 flow cytometry assay. The positive control group was obtained by treating HTR-8/SVneo cells with CCCP at the ratio of 1:1000 for 20 min. Data were analyzed using FlowJo software and presented as means ± SEM (n = 3/group). Statistical comparisons were conducted utilizing one-way ANOVA with the Tukey post hoc test. * *p* < 0.05, *** *p* < 0.001, **** *p* < 0.0001 in comparison to the control group. Notes: ANOVA, analysis of variance; CCCP, carbonyl cyanide 3-chlorophenylhydrazone; MMP (ΔΨm), mitochondrial membrane potential; PS-MPs, polystyrene microplastics; PS-MNPs, polystyrene micro/nanoplastics; PS-NPs, polystyrene nanoparticles; SEM, standard error of mean.

In trophoblast cellular functional assessment, we observed an increase in early apoptosis following exposure to PS-NPs. Mitochondria play a pivotal role in the apoptotic process, with mitochondrial membrane potential (ΔΨm) reduction correlating with the early apoptosis cascade. Therefore, we conducted a ΔΨm assay on HTR8/SVneo cells exposed to 50 nm, 200 nm, and 2 μm PS-MNPs under high-dose conditions using JC-1 dye, with the cccp-treated group serving as a positive control. Our results indicate a significant reduction ΔΨm in trophoblast cells exposed to high concentrations of 50 nm and 200 nm PS-NPs (* *p* < 0.05, *** *p* < 0.001; Figure 5A–D and Figure S3A–D), while exposure to 2 μm PS-MPs showed no significant impact on ΔΨm (Figure 5E,F and Figure S3E,F).

### 3.7. Comprehensive Assessment of PS-MNP’s Placental Toxicity with the TRACE Strategy

We have devised a novel environmental chemical risk assessment strategy, conducting a comprehensive evaluation of reliability, relevance, and outcome metrics for both existing literature evidence and our own research. The scoring was independently conducted by two researchers, and discrepancies were resolved and analyzed by two additional researchers to ensure the validity of the results. The calculation formula is as follows:

$$\mathrm{Comprehensive}\;\mathrm{Evidence}\;\mathrm{Score}=\;\frac{\Sigma (Reliability\;Score\times Correlation\;Score\times Outcome\;Fitness\;score)}{Evidence\;Number}\;\times \;\mathrm{Integrity}\mathrm{scores}.$$

**Figure 6:**
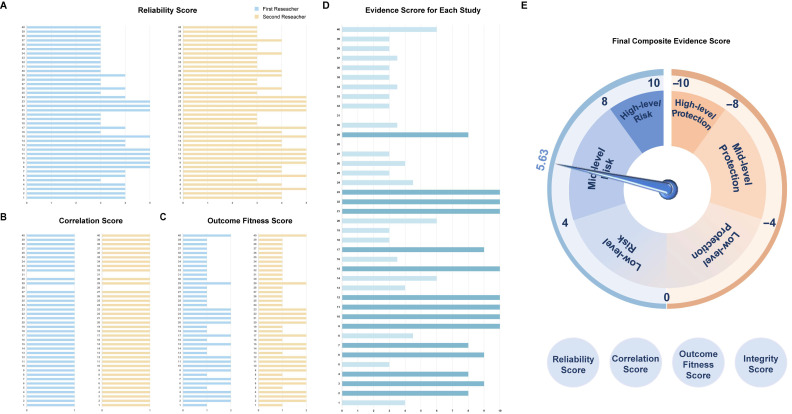
The scoring results of the two researchers for all the evidence: (**A**) Results of the reliability score; (**B**) Results of the correlation score; (**C**) Results of outcome fitness score; (**D**) Final composite evidence scores for each study; (**E**) Composite evidence scores for all the studies. The horizontal axis represents scores, and the vertical axis depicts the quantity of studies. The final studies included in the analysis were those listed in Table 1, Table 2 and Table 3, as well as the researcher’s own studies.

Reliability Scores: The study design and execution were assessed on a scale of 1 to 5 points based on their quality. The reliability scores of the two researchers for all the evidence (Figure 6A).

Correlation Scores: The relationship between the exposure and outcome observed in the study had a positive correlation score of 1 point, a negative correlation score of −1 point, and no significant correlation score of 0 points. The correlation scores of the two researchers for all the evidence (Figure 6B).

Outcome Fitness Scores: The consistency between the study outcomes and the overarching hypothesis is assessed on a scale of 1 to 2 points. The outcome fitness scores of the two researchers for all the evidence (Figure 6C).

Evidence Number: The total number of items of evidence involved in scoring.

Integrity Scores: The integrity score is divided equally among in vivo, in vitro, and epidemiological evidence, each accounting for one-third. In this study, integrity scores total 100%.

Comprehensive Evidence Score: The overall validity, directionality, and strength of association representing all evidence. The comprehensive evidence scores of the two researchers for all the evidence (Figure 6D).

Of particular note is that in instances where a publication encompasses two or more study types, we categorize them separately for analysis and evaluation.

A risk matrix adopting the coefficients described above was designed, thus classifying the health risks of the target chemicals as low, medium, or high: (0, 4] as low-level risk, (4, 8] as medium-level risk, and (8, 10] as high-level risk; (0, −4] as low-level protection, (−4, −8] as medium-level protection, and (−8, −10] as high-level protection (Figure S4). After integrating statistical calculations, the final score was 5.63, indicating that MNPs pose a moderate risk of reproductive toxicity to the placenta (Figure 6E).

## 4. Discussion

MNPs are emerging as potential threats to reproductive health in studies regarding the environment and human health [57,58]. Direct evidence linking this risk to humans is currently lacking. Placental disorders during pregnancy may be attributed to adverse environmental exposure by expectant mothers before or during gestation [59,60,61]. The viability, proliferation, and migration abilities of trophoblast cells are crucial for normal embryonic development, and adverse environmental exposures before or during pregnancy may cause detrimental changes in these phenotypes, potentially contributing to placental diseases. Accumulation of MNPs has been detected in the placenta [20], meconium [21], and amniotic fluid [11], yet the potential risks to both pregnant women and fetuses remain unconfirmed. Our comprehensive review of the publications on MNPs and placental reproductive toxicity indicates an inverse correlation between toxicity and particle size. Using human extravillous trophoblast cells, we investigated the functional repercussions of MNP exposure across varying particle sizes, thus corroborating this finding. The objective of this study is to evaluate the risk levels of MNP exposure to placental reproductive toxicity by applying an innovative environmental chemical risk assessment strategy, offering valuable insights into the association between prenatal microplastic exposure and adverse pregnancy outcomes.

MNPs have attracted more attention than environmental chemical substances due to their variable particle sizes and additional physical properties. Currently, particles, including MNPs, have been found in normal tissues and body fluids, with higher accumulations in disease samples such as feces from inflammatory bowel disease patients [62], lung cancer [63], and liver cirrhosis [10] specimens. However, causal relationships between microplastic exposure and diseases largely elude determination, given these findings are predominantly observational. Only a recent study demonstrated the accumulation of MNPs in arteries elevates the risk of cardiovascular disease by 3.5 times [18]. Due to population biases in epidemiological data and other confounding factors, elucidating causality between microplastics and cardiovascular diseases proves challenging in this study. The judgment of the MNP’s harmful effect on human health relies largely on extrapolation from in vitro or in vivo experiments, owing to the lack of evidence regarding their toxicity in the human body [64,65,66]. Indeed, the experimental doses of MNPs often surpass environmental levels, leading to an exaggerated overestimation in animal or cell models [67,68]. The unique physicochemical properties and variable particle sizes of microplastics, coupled with the complexity of population exposures and limitations in detection techniques, mean that there is presently no reasonable dose conversion relationship to translate actual human exposure levels into experimental doses. It is necessary to develop a more scientifically rigorous, cost-effective, and comprehensive strategy for assessing the toxicity of MNPs.

Adverse Outcome Pathways (AOPs) are chemical risk management strategies initiated by the Organization for Economic Co-operation and Development (OECD) in 2010, stemming from the mechanisms of toxicity [69,70,71]. They link molecular initiating events to adverse outcomes, establishing logical connections between toxic features and molecules, cells, tissues, organs, individuals, and populations. AOPs function as conceptual frameworks guiding the direction of toxicity research on environmental chemicals, although their complexity and high cost remain significant challenges [72,73]. Other internationally available toxicity evaluation systems, including a software-based tool (ToxRTool, version 2009) developed by a European Commission project that provides guidance on the reliability assessment of toxicological data, and the Science in Risk Assessment and Policy (SciRAP) have enhanced risk assessment transparency and structural standardization [74,75]. However, these tools were considered suitable for evidence assessment in vivo and in vitro experiments. A meta-analysis combines and synthesizes the results of multiple independent studies to derive a broader and statistically significant conclusion, but it is not applicable to risk assessment. We strive to develop a straightforward yet rigorous approach for promptly comprehending the escalating risks associated with emerging environmental chemicals. This foundational understanding will empower us to delineate precise research directions and evaluate the accompanying risks.

Our innovative TRAEC strategy fully leveraged published and emerging evidence and comprehensively integrated various research data to provide a new standardized process for chemical toxicity assessment. This study employed the TRAEC strategy with multidimensional scoring, indicating that the placental reproductive toxicity of MNPs was at a moderate-to-low risk level, with a score of 5.63. We conducted cell viability assays on three sizes of PS-MNPs, showing a dose-dependent decrease in cell viability after exposure to 50 nm PS-NPs for 24 and 48 h. Exposure to 200 nm PS-NPs for 48 h also affected cell viability. Embryonic development depends on the normal viability and proliferation of trophoblast cells. Under specific conditions, such as the presence of reproductive system tumors, abnormal cell proliferation can occur, as observed in our study, where exposure to 2 μm PS-MPs led to increased activity in the CCK-8 assay. Furthermore, our original data from assays indicated that HTR-8/SVneo cells exposed to 50 nm NPs exhibited greater functional impairments, including decreased migration and invasion abilities, increased apoptosis, and reduced mitochondrial membrane potential, compared to those exposed to 200 nm NPs under the same conditions. Conversely, cell functions were not significantly altered upon exposure to 2 μm MPs under the same conditions. We hypothesized that plastic particles with smaller diameters may be more readily internalized into cells, eliciting relatively higher cellular toxicity.

The TRAEC strategy served as an efficient risk assessment guideline developed by our team to address the complexities and variations inherent in environmental chemicals. However, it had certain limitations in evaluating the toxicity of MNPs. Firstly, the intricate physicochemical properties of MNPs allowed them to act as carriers for adsorbing various environmental chemicals, potentially leading to their simultaneous entry into the human body [76]. This necessitates further exploration of composite toxicity assessment. Plastic particles of similar sizes may yield inconsistent toxicological responses across different types of studies, prompting the establishment of distinct standardized scoring criteria for various types of literature. Secondly, a requisite scientific foundation was necessary to enable the multiple rounds of screening and scoring of published literature, focusing on the relationship between exposure substances and outcomes under investigation. Discrepancies between experimental doses and actual exposure doses in the population often result in discrepant target outcomes in exposure studies of varying types. Hence, we endeavor to develop more refined scoring criteria to address this issue.

## 5. Conclusions

This study proposed a novel environmental chemical risk assessment approach to investigate the relationship between maternal exposure to MNPs and adverse pregnancy outcomes, integrating existing scientific evidence with our original experimental data. Our findings indicated that the placental reproductive toxicity of MNPs was at a low-to- moderate risk level according to the TRAEC strategy. At high exposure levels, smaller plastic particles exhibited greater potential reproductive toxicity, suggesting a dose- and particle-size-dependent effect of MNPs. Overall, the TRAEC strategy integrated published epidemiologic, in vivo, and in vitro evidence with our original data from assays to provide a comprehensive rationale for assessing the risks of MNP exposure during pregnancy. This strategy enhances our understanding of the potential effect of ubiquitous MNPs on the reproductive health of pregnant women.

## Data Availability

Data is contained within the article or Supplementary Material.

## Supplementary Materials

The following supporting information can be downloaded at: https://www.mdpi.com/article/10.3390/toxics12080553/s1, Figure S1: HTR-8/SVneo cells were exposed to 2 μm PS-MPs; Figure S2: Changes in the viability of HTR-8/SVneo cells following a 12-hour exposure to PS-MNPs; Figure S3: The mitochondrial membrane potential changes in HTR-8/SVneo cells exposed to low doses of PS-MNPs; Figure S4: The risk matrix and risk classification were established in accordance with the TRAEC strategy framework.
