# Supplementary material for: Micro/Nanoplastic Exposure on Placental Health and Adverse Pregnancy Risks: Novel Assessment System Based upon Targeted Risk Assessment Environmental Chemicals Strategy
Source: Toxics. 2024 Jul 30;12(8):553. doi: 10.3390/toxics12080553 (PMC11359514; doi:10.3390/toxics12080553)
Supplement: Supplementary file 1 [file toxics-12-00553-s001.zip › toxics-3094745-supplementary.pdf]

toxics

MDPI

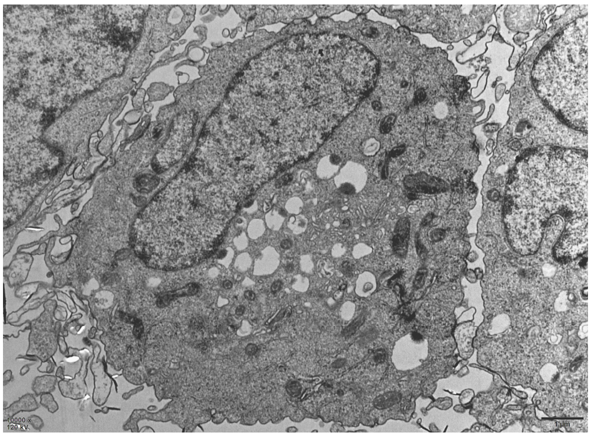

Figure S1. HTR-8/SVneo cells were exposed to 2 µm PS-MPs. (A) The representative transmission electron microscopy image of HTR-8 /SVneo cells treated with 2 µm PS-MPs at a concentration of 100 µg/mL for 48 h. Note: PS-MPs, polystyrene microparticles; TEM, transmission electron microscope.

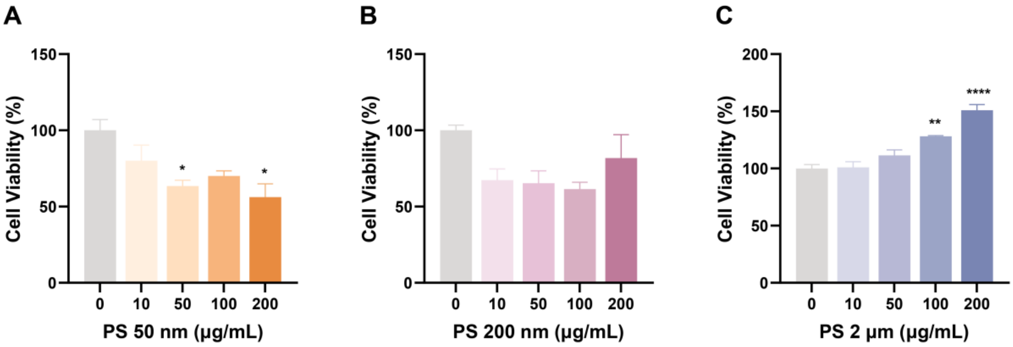

Figure S2. Changes in the viability of HTR-8/SVneo cells following a 12-hour exposure to PS-MNPs. (A-C) The proliferation capacity of HTR-8/SVneo cells was detected by CCK-8 12 h exposure to PS-MNPs of 50 nm (A), 200 nm (B), and 2 µm (C) at different concentrations (0, 10, 50, 100, 200 µg/mL). Results were presented as means ± SEM (n=3/group). Statistical comparisons were performed using one-way ANOVA with the Tukey post hoc test. *$p < 0.05$, **$p < 0.01$, ****$p < 0.0001$ in comparison to the control group (0 µg/mL). Note: ANOVA, analysis of variance; CCK-8, cell counting kit-8; PS-MNPs, polystyrene micro-/nano-plastics; PS-NPs, polystyrene nanoparticles; SEM, standard error of mean.

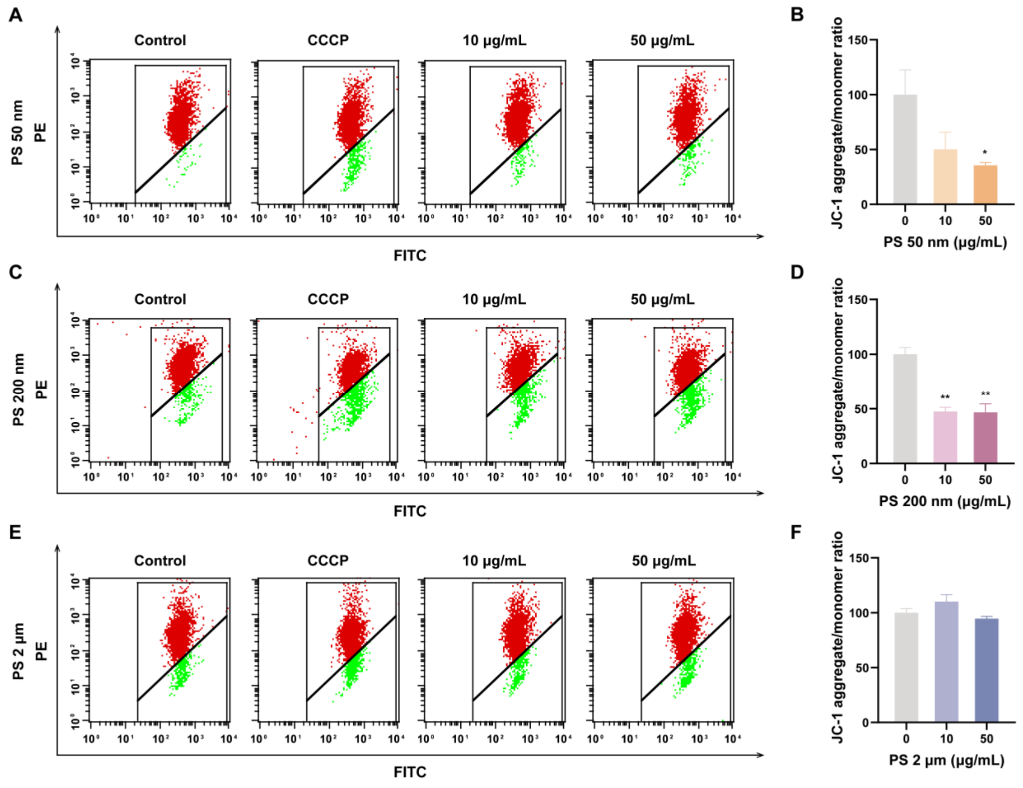

Figure S3. The mitochondrial membrane potential changes in HTR-8/SVneo cells exposed to low doses of PS-MNPs. (A-B) MMP (ΔΨm) of trophoblast cells was assessed using flow cytometry after staining with JC-1 after exposure to 0 (Control), 10, 50 μg/mL 50 nm PS-NPs for 48 h; (C-D) The analysis of MMP (ΔΨm) using JC-1 Dye conducted via flow cytometry. The cells were exposed to different concentrations of 200 nm PS-NPs (0 μg/mL as control, 10 μg/mL, and 50 μg/mL) for a duration of 48 h; (E-F) The MMP (ΔΨm) of HTR-8/SVneo cells was assessed using the JC-1 flow cytometry assay after being treated with 2 μm PS-MPs at concentrations of 0 (Control), 10, and 50 μg/mL. The positive control group was obtained by treating HTR-8/SVneo cells with CCCP at the ratio of 1:1000 for 20 min. Data were analyzed using FlowJo software and presented as means ± SEM (n=3/group). Statistical comparisons were conducted utilizing one-way ANOVA with the Tukey post hoc test. *$p < 0.05$, **$p < 0.01$ in comparison to the control group. Notes: ANOVA, analysis of variance; CCCP, carbonyl cyanide 3-chlorophenylhydrazone; MMP (ΔΨm), mitochondrial membrane potential; PS-MPs, polystyrene microplastics; PS-MNPs, polystyrene micro-/nano-plastics; PS-NPs, polystyrene nanoparticles; SEM, standard error of mean.

Correlation Fit Scores / Reliability Scores	The potential health risk level of environmental compounds (scores)			
	**Very likely negative correlation (−2)**	**Possible negative correlation (−1)**	**Possible positive correlation (1)**	**Very likely positive correlation (2)**
**Extremely High (4,5]**	High-Protection (−8,−10]	Mid-Protection (−4,−5]	Mid-Risk (4,5]	High-Risk (8,10]
**High (3,4]**	Mid-Protection (−6,−8]	Low-Protection (−3,−4]	Low-Risk (3,4]	Mid-Risk (6,8]
**Medium (2,3]**	Mid-Protection (−4,−6]	Low-Protection (−2,−3]	Low-Risk (2,3]	Mid-Risk (4,6]
**Low (1,2]**	Low-Protection (−2,−4]	Low-Protection (−1,−2]	Low-Risk (1,2]	Low-Risk (2,4]
**Extremely Low (0,1]**	Low-Protection (0,−2]	Low-Protection (0,−1]	Low-Risk (0,1]	Low-Risk (0,2]

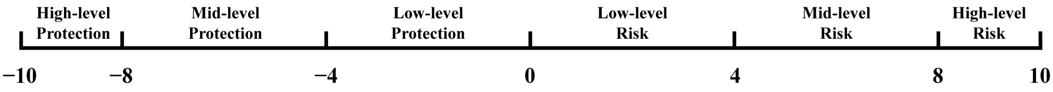

Figure S4. The risk matrix and risk classification were established in accordance with the TRAEC strategy framework.
